# Mutant KRAS-Associated Proteome Is Mainly Controlled by Exogenous Factors

**DOI:** 10.3390/cells11131988

**Published:** 2022-06-21

**Authors:** Patrícia Dias Carvalho, Flávia Martins, Joana Carvalho, Maria José Oliveira, Sérgia Velho

**Affiliations:** 1 Instituto de Investigação e Inovação em Saúde (i3S), Rua Alfredo Allen 208, 4200-135 Porto, Portugal; pcarvalho@ipatimup.pt (P.D.C.); flaviam@ipatimup.pt (F.M.); jcarvalho@ipatimup.pt (J.C.); mariajo@ineb.up.pt (M.J.O.); 2 Institute of Molecular Pathology and Immunology, University of Porto (IPATIMUP), Rua Júlio Amaral de Carvalho 45, 4200-135 Porto, Portugal; 3 Institute of Biomedical Sciences Abel Salazar (ICBAS), University of Porto, R. Jorge de Viterbo Ferreira 228, 4050-313 Porto, Portugal; 4 Department of Pathology, Faculty of Medicine, University of Porto (FMUP), Alameda Prof. Hernâni Monteiro, 4200-319 Porto, Portugal; 5 Institute of Biomedical Engineering (INEB), University of Porto, Rua Alfredo Allen, 4200-135 Porto, Portugal

**Keywords:** mutant KRAS, colorectal cancer, cancer-associated fibroblasts, proteomic analysis

## Abstract

Understanding how mutant KRAS signaling is modulated by exogenous stimuli is of utmost importance to elucidate resistance mechanisms underlying pathway inhibition failure, and to uncover novel therapeutic targets for mutant KRAS patients. Hence, aiming at perceiving KRAS-autonomous versus -non autonomous mechanisms, we studied the response of two mutant KRAS colorectal cancer cell lines (HCT116 and LS174T) upon KRAS silencing and treatment with rhTGFβ1-activated fibroblasts secretome. A proteomic analysis revealed that rhTGFβ1-activated fibroblast-secreted factors triggered cell line-specific proteome alterations and that mutant KRAS governs 43% and 38% of these alterations in HCT116 and LS174T cells, respectively. These KRAS-dependent proteins were localized and displayed molecular functions that were common to both cell lines (e.g., extracellular exosome, RNA binding functions). Moreover, 67% and 78% of the KRAS-associated proteome of HCT116 and LS174T cells, respectively, was controlled in a KRAS-non-autonomous manner, being dependent on fibroblast-secreted factors. In HCT116 cells, KRAS-non-autonomously controlled proteins were mainly involved in proteoglycans in cancer, p53, and Rap1 signaling pathways; whereas in LS174T cells, they were associated with substrate adhesion-dependent cell-spreading and involved in metabolic processes. This work highlights the context-dependency of KRAS-associated signaling and reinforces the importance of integrating the tumor microenvironment in the study of KRAS-associated effects.

## 1. Introduction

KRAS belongs to the RAS family of small GTPases. Owing to its inner membrane location, KRAS functions as a transducer of extracellular stimuli to the interior of the cell. By integrating signals from different tyrosine kinase receptors (RTKs), KRAS governs several distinct signaling cascades, such as RAF–MEK–ERK, PI3K–AKT–mTOR, and RALGDS–RAL, dictating cell fate. Missense substitutions occurring in *KRAS* gene, either reduce GTP hydrolysis or increase the rate of GTP loading, altering its ON/OFF homeostasis towards the active state, thus driving cell proliferation and survival [1,2].

Standing as the most frequently mutated oncogene in human cancer, with particular relevance in pancreatic, colorectal (CRC) and lung cancers [2], KRAS is considered a key therapeutic target. However, despite of extensive attempts to target KRAS or its downstream signaling effectors, only recently a KRAS G12C specific inhibitor, Sotorasib (Lumakras, from Amgen), demonstrated clinical efficiency [3]. Notwithstanding this major therapeutic breakthrough, a phase 2 clinical trial with Sotorasib in previously treated CRC patients, showed that only 6 out of 62 patients displayed partial response [4]. In addition to intrinsic resistance, other studies have also highlighted a high percentage of acquired resistance to KRAS G12C inhibition [5]. So, one must interrogate why so many different drugs and strategies show limited therapeutic efficiency when reaching the clinical setting. Despite the alterations induced by specific mutations, mutant KRAS (mutKRAS) forms still depend, to a certain extent, on the activation by external stimuli [6,7]. Moreover, different mutations display distinct effector affinities and levels of activation, dictating diverse effects [2,8,9,10]. Likewise, KRAS downstream effects have been reported to be context-dependent, showing allele and tissue/tumor specificities [11,12]. In addition, it has been demonstrated that signaling heterogeneity in both the tumor and microenvironment impacts treatment response [13]. Therefore, the study of the crosstalk of mutKRAS cancer cells with the tumor microenvironment (TME) components acquires a special relevance. In fact, mutKRAS cancer cells have been shown to communicate with and to modulate the TME, favoring tumor progression and malignancy [14,15,16,17,18]. As so, in vitro studies evaluating drug responses only considering cancer cells in their optimal culture conditions, are very reductionist and might not generate translational knowledge. Thus, more integrative studies are needed to better understand the impact of the microenvironment on KRAS-driven signaling and therapy response/resistance. In line with this, our group has recently shown that mutKRAS can regulate functional effects autonomously or it can cooperate with fibroblast-secreted factors to modulate cancer cell invasive behavior [19].

Herein, we aimed to demonstrate the impact of microenvironmental cues on KRAS-driven signaling. As fibroblasts are one of the major components of the TME, we used rhTGFβ1-activated fibroblast-derived secretome as a source of microenvironment signals. We demonstrated that mutKRAS controls both autonomous, and non-autonomous fibroblast-dependent signaling. Noteworthy, we showed that 2/3 of the total proteome regulated by mutKRAS are stimuli-dependent. Moreover, fibroblast-derived signals even reversed the expression trend of some KRAS-regulated proteins when comparing stimulated with non-stimulated cells. Overall, our data show that the mutKRAS-associated proteome profile drastically changes in response to external stimulation, suggesting that its oncogenic signaling is mainly regulated in a non-autonomous manner. As such, we propose that studies addressing the oncogenic effects of KRAS, the identification of therapeutic targets or biomarkers of therapy resistance should take into consideration the influence of the microenvironment in dictating KRAS signaling.

## 2. Materials and Methods

### 2.1. Cell Culture

HCT116 colorectal cancer cell line and CCD-18Co normal colon fibroblasts were purchased from American Type Culture Collection (ATCC). LS174T cells were kindly provided by Dr. Ragnhild A. Lothe (Oslo University Hospital, Oslo, Norway). HCT116 cells were cultured in RPMI 1640 medium (Gibco, Thermo Fisher Scientific, Waltham, MA, USA) and LS174T and CCD-18Co were cultured in DMEM medium (Gibco, Thermo Fisher Scientific, Waltham, MA, USA). For all cell lines the respective medium was supplemented with 10% fetal bovine serum (Hyclone, Logan, UT, USA) and 1% penicillin–streptomycin (Gibco, Thermo Fisher Scientific, Waltham, MA, USA). Cells were maintained at 37 °C in a humidified atmosphere with 5% CO_2_.

### 2.2. CCD-18Co Conditioned Media Production

For conditioned media (CM) production, the same number of cells was plated in two T75 culture flasks and cultured until approximately 90% of confluence. At the desired confluence, cells were washed twice with PBS buffer and new media was added. “Normal-like” fibroblasts were cultured in serum-free DMEM (supplemented only with 1% penicillin/streptomycin) and “activated-fibroblasts” were cultured in the same medium supplemented with 10 ng/mL of rhTGFβ1 (ImmunoTools GmbH, Friesoythe, Germany) (FibCM). As control media, DMEM+1% penicillin/streptomycin and DMEM+1% penicillin/streptomycin+10 ng/mL rhTGFβ1 (ctrlCM) were added to culture flasks without cells. After four days in optimal culture conditions, CM were harvested, centrifuged, filtered through a 0.2 μm filter and stored at −20 °C until use. Cells were trypsinized and counted to assure an equivalent number of cells in both conditions. Total protein was extracted, and fibroblast-activation was confirmed through the evaluation of alpha smooth muscle actin (α-SMA) expression by Western blot (Figure S1). 

### 2.3. Gene Silencing by siRNA Transfection and Treatment with Conditioned Media

Cells were seeded in six-well plates (150,000 and 200,000 cells for HCT116 and LS174T, respectively) and transfected after approximately 16 h, using Lipofectamine RNAiMAX (Invitrogen, Thermo Fisher Scientific, Waltham, MA, USA) in reduced-serum Opti-MEM medium (Gibco, Thermo Fisher Scientific), following manufacturer’s instructions. Gene silencing was achieved with ON-TARGETplus SMARTpool small interfering RNA specific for KRAS (L-005069-00-0010; Dharmacon, Lafayette, CO, USA) at a final concentration of 10 nM. A non-targeting siRNA (D-001810-01-50; ON-TARGETplus Non-targeting siRNA #1, Dharmacon, Lafayette, CO, USA) was used as a negative control. Seventy-two hours after transfection, control (siCTRL) and KRAS silenced (siKRAS) HCT116 and LS174T cells, were washed and treated with serum free CM from rhTGFβ1-activated fibroblasts (FibCM) and the respective control (ctrlCM), during 24 h. KRAS silencing efficiency was monitored by Western blot (Figure S2). For each cell line, three independent biological replicates were performed.

### 2.4. Protein Extraction and Western Blotting

Total protein was extracted using ice cold RIPA Buffer [25 mM Tris-HCl pH = 7–8; 150 mM NaCl; 0.5% sodium deoxycholate; 1% triton X-100] supplemented with a protease inhibitor cocktail (Roche, Basel, Switzerland) and a phosphatase inhibitor cocktail (Sigma-Aldrich, St. Louis, MO, USA). Protein concentration was determined using the DCProtein assay kit from BioRad (Hercules, CA, USA) and, from the cells treated with CM, 100 µg of total protein were processed for proteomics analysis. Furthermore, 25 µg (in the case of CRC cell lines) or 15 µg (in the case of CCD-18Co fibroblasts) of protein were resolved on sodium dodecyl sulphate-polyacrylamide gel electrophoresis (SDS-PAGE) under denaturing conditions and transferred to Protran Premium NC 0.45 µm membranes (Amersham Biosciences, GE Healthcare, Cardiff, UK). Membranes were blocked for 1h at RT and incubated overnight at 4 °C with agitation, with the respective primary antibody against KRAS (LS-Bio, LS-C175665; 1:4000), α-SMA (Abcam, Cambridge, UK; ab7817, 1:250) or GAPDH (Santa Cruz Biotechnology, Dallas, TX, USA; sc-47724, 1:10,000), all diluted in 5% non-fat milk in PBS+ 0.5% Tween 20. After incubation with the specific anti-mouse (NA931, GE Healthcare) HRP-conjugated secondary antibody for 1h at RT, bands were detected using ECL (BioRad, Hercules, CA, USA) and film sheets exposure (Amersham Biosciences, GE Healthcare, UK). 

### 2.5. Sample Processing for Proteomic Analysis 

Sample processing and proteomic analysis were performed at i3S Proteomics Scientific Platform.

For proteomic analysis, a single-step reduction and alkylation with tris-2(-carboxyethyl)-phosphine (TCEP)/chloroacetamide (CAA) was performed in combination with the single-pot solid-phase-enhanced sample preparation (SP3) protocol, as described elsewhere [20,21]. Briefly, to reduce disulfide bonds and alkylate cysteines, sodium deoxycholate (SDC) 2x buffer (200 mM Tris pH 8.5, SDC 2%, 20 mM TCEP, 80 mM CAA) was added to the protein sample and incubated for 10 min at 95 °C, 1000 rpm. Next, 100 µL of Sera-Mag Magnetic Beads (10 µg/µL, GE Healthcare, Chicago, IL, USA) and ethanol 100% were added to the protein solution (50% ethanol, final concentration), resuspended, and incubated at 22 °C for 10 min at 1000 rpm. After complete binding, samples were washed with 80% ethanol. To achieve protein enzymatic digestion, 50 µL of triethylammoniumbicarbonate (TEAB) 50 mM mixed with Trypsin + LysC (2 µg) was added to the beads and incubated overnight at 37 °C with 1000 rpm agitation. In the next day, 1.3 mL of 100% acetonitrile (ACN) was added to the samples and incubated for 20 min at 22 °C, 1000 rpm. Tubes were placed in a magnetic rack and beads were washed with of 100% ACN. Following, 100 µL of TEAB 50 mM was added to the beads, resuspended, and incubated for 5 min at 22 °C, 1000 rpm. After incubation, tubes were placed in a magnetic rack until the beads have migrated to the tube wall, and the supernatant was transferred to a new tube with 20 µL of 5% formic acid (FA). Afterwards, tubes with FA were placed in a SpeedVac until the samples were dry. Then, samples were resuspended in 0.1% FA and peptides were quantified by Pierce™ Quantitative Fluorometric Peptide Assay (Thermo Fisher Scientific, Waltham, MA, USA). Five hundred nanograms of peptides of each sample were analyzed by Liquid Chromatography-Mass Spectrometry (LC-MS).

### 2.6. LC-MS/MS-Analysis

Protein identification and quantitation were performed by nanoLC–MS/MS using an Ultimate 3000 liquid chromatography system coupled to a Q-Exactive Hybrid Quadrupole-Orbitrap mass spectrometer (Thermo Fisher Scientific, Bremen, Germany), following published protocols [22]. Specifically, samples were loaded onto a trapping cartridge (Acclaim PepMap C18 100 Å 5 mm × 300 µm i.d., 160454, Thermo Fisher Scientific, Bremen, Germany) in a mobile phase of 2% ACN, 0.1% FA at 10 µL/min. After 3 min loading, the trap column was switched in-line to a 50 cm by 75 μm inner diameter EASY-Spray column (ES803, PepMap RSLC, C18, 2 μm, Thermo Fisher Scientific, Bremen, Germany) at 300 nL/min. Separation was generated by mixing A: 0.1% FA, and B: 80% ACN, with the foll@owing gradient: 5 min (2.5% B to 10% B), 120 min (10% B to 30% B), 35 min (30% B to 50% B), 3 min (50% B to 99% B), and 12 min (hold 99% B). Data acquisition was controlled by Xcalibur 4.0 and Tune 2.9 software (Thermo Fisher Scientific, Bremen, Germany).

The mass spectrometer was operated in data-dependent (dd) positive acquisition mode alternating between a full scan (m/z 380–1580) and subsequent HCD MS/MS of the 10 most intense peaks from full scan (normalized collision energy of 27%). ESI spray voltage was 1.9 kV. The following settings were used: global settings—use lock masses best (m/z 445.12003) and lock mass injection Full MS, chrom. peak width (FWHM) 15 s; full scan settings—70 k resolution (m/z 200), AGC target 3 × 10^6^, maximum injection time 120 ms; dd settings—minimum AGC target 8 × 10^3^, intensity threshold 7.3 × 10^4^, charge exclusion: unassigned, 1, 8, >8, peptide match preferred, exclude isotopes on, and dynamic exclusion 45 s; and MS2 settings—microscans 1, resolution 35 k (m/z 200), AGC target 2 × 10^5^, maximum injection time 110 ms, isolation window 2.0 m/z, isolation offset 0.0 m/z, and spectrum data type profile.

### 2.7. Data and Bioinformatics Analysis

The acquired raw data were analyzed using the Proteome Discoverer 2.5.0.400 software (Thermo Scientific, Bremen, Germany) and searched against the UniProt database for the *Homo sapiens* Proteome 2020_05 (75,069 entries). The Sequest HT search engine was used to identify tryptic peptides. The ion mass tolerance was 10 ppm for precursor ions and 0.02 Da for fragment ions. Maximum allowed missing cleavage sites was set to 2. Cysteine carbamidomethylation was defined as constant modification. Methionine oxidation, serine, threonine and tyrosine phosphorylation and protein N-terminus acetylation were defined as variable modifications. Peptide confidence was set to high. The processing node Percolator was enabled with the following settings: maximum delta Cn 0.05; decoy database search target FDR 1%, validation based on q-value. Protein label free quantitation was performed with the Minora feature detector node at the processing step. Precursor ions quantification was performing at the processing step with the following parameters: unique plus razor peptides were considered for quantification, precursor abundance was based on intensity, normalization mode was based on total peptide amount, protein ratio calculation was pairwise ratio based, imputation was not performed, and hypothesis test was based on t-test (background based). The mass spectrometry proteomics data were deposited to the ProteomeXchange Consortium via the PRIDE [23] partner repository with the dataset identifier PXD030551 and 10.6019/PXD030551. Lists with all proteins identified for each cell line (Supplementary Files S1 and S2) and the result of each comparison (Supplementary Files S3–S8) are included as supplementary files.

Gene Ontology (GO) analysis and Kyoto Encyclopedia of Genes and Genomes (KEEG) pathway analysis was performed using DAVID Bioinformatics. Differentially expressed proteins were selected by applying the following criteria as cutoff: Abundance Ratio Adj. *p*-value less than or equal to 0.05 and unique peptides greater than or equal to 2. Upregulated proteins were defined according to an abundance ratio greater than or equal to 2; whilst downregulated proteins displayed an abundance ratio lower than or equal to 0.5. Graphics were made using GraphPad Prism version 9.0.0 and the enrichment score was calculated by—log *p*-value.

## 3. Results

To investigate the impact of fibroblast-derived microenvironmental factors on KRAS-driven effects, we profiled the total proteome of two distinct mutKRAS CRC cell lines (HCT116 and LS174T) upon KRAS silencing followed by treatment with CM from rhTGFβ1-activated fibroblasts (siKRAS_FibCM). As controls, we used cells transfected with a non-targeting siRNA (siCTRL) cultured with control and fibroblasts CM (siCTRL_ctrlCM and siCTRL_FibCM, respectively), and KRAS silenced (siKRAS) cells cultured with control CM (siKRAS_ctrlCM). FibCM was derived from normal-like colon fibroblast cell line (CCD-18Co) activated with rhTGFβ1 to mimic cancer-associated fibroblasts (CAFs) phenotype [24,25,26]. All proteomics data were deposited to the ProteomeXchange Consortium and freely available with identifier PXD030551 and 10.6019/PXD030551. Summarized data are included as supplementary (Supplementary Files S1–S10).

### 3.1. Fibroblast-Secreted Factors Impact the Proteome of CRC Cell Lines

First, we analyzed the overall impact of fibroblasts-secreted factors on the proteome of HCT116 and LS174T CRC cell lines. To do so, we compared the protein expression profiles of siCTRL cells cultured in FibCM or in ctrlCM. 

**Figure 1:**
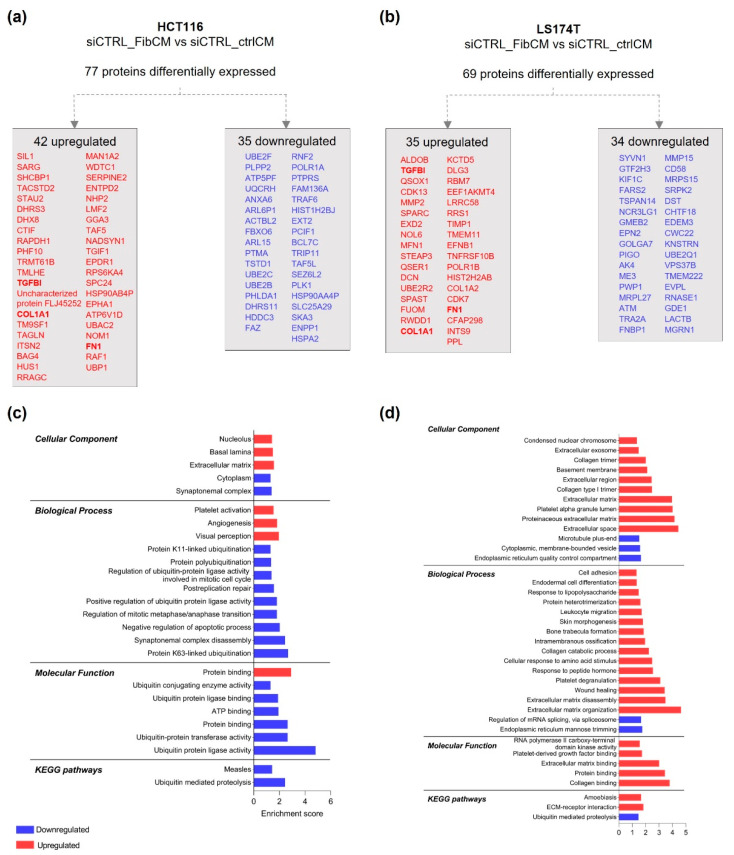
**HCT116 and LS174T differentially expressed proteins, upon culture with rhTGFβ1-activated fibroblasts conditioned medium.** Significantly up and downregulated proteins in HCT116 (**a**) and LS174T (**b**) siCTRL cells cultured with FibCM (siCTRL_FibCM vs. siCTRL_ctrlCM). Protein lists are organized according to the abundance ratio and common proteins to HCT116 and LS174T cells are highlighted in bold (TGFBI, COL1A1, FN1). GO and KEGG pathways analysis of up and downregulated proteins in HCT116 (**c**) and LS174T (**d**). The enrichment score was calculated by—log *p*-value.

HCT116 cells displayed 77 differentially expressed proteins, with 42 upregulated and 35 downregulated upon treatment with FibCM (Figure 1a and Supplementary File S9). The 42 upregulated proteins were localized at the nucleolus, at the basal lamina, and at the extracellular matrix. These proteins were mainly involved in angiogenesis and exhibited protein binding functions. The 35 downregulated proteins were mostly localized at the cytoplasm, and were involved in several ubiquitination processes, negative regulation of apoptosis, and processes involved in cell division (Figure 1c).

LS174T cells presented 69 differentially expressed proteins, with 35 upregulated and 34 downregulated upon treatment with FibCM (Figure 1b and Supplementary File S10). The 35 upregulated proteins were mostly associated with extracellular components, including: extracellular matrix, extracellular space, extracellular exosome, and basement membrane. These proteins spanned across several biological processes regarding ECM disassembly/organization, wound healing, collagen catabolic process, and cell adhesion and were involved in ECM-receptor interactions. Accordingly, they displayed collagen, protein, extracellular matrix, and platelet-derived growth factor binding functions, and RNA polymerase II carboxy-terminal domain kinase activity. The 34 downregulated proteins were localized at the endoplasmic reticulum quality control compartment, at the cytoplasmic membrane-bounded vesicles, and at the microtubule plus-end. They were involved in mRNA splicing regulation and endoplasmic reticulum mannose trimming as well as in ubiquitin mediated proteolysis pathway (Figure 1d).

Interestingly, GO analysis revealed that HCT116 and LS174T cells treated with FibCM overexpressed common proteins associated with the extracellular matrix, namely: Collagen Type I Alpha 1 (COL1A1), Fibronectin (FN1), and Transforming Growth Factor Beta Induced protein (TGFBI) (Figure 1a,b). Likewise, KEGG analysis revealed that both cell lines displayed downregulated proteins involved in ubiquitin mediated proteolysis pathways (Figure 1c,d).

Together these data show that TGFβ1-activated fibroblasts secretome alters CRC cells proteome, mainly in a cell line-dependent manner.

### 3.2. MutKRAS Modulates the Proteomic Profile Associated with Cancer Cell Response to Fibroblast-Secreted Factors

Functioning as a downstream target of several cell surface receptors, KRAS is a major signaling hub, bridging external cues and internal signaling pathways. Thus, upon showing that fibroblast-secreted factors modulate the protein profile of CRC cells, we next sought to determine whether mutKRAS orchestrates the cell response to fibroblast-secreted factors. To do so, we compared the expression profiles previously obtained in the siCTRL_FibCM vs. siCTRL_ctrlCM analysis with the expression profiles of siKRAS vs. siCTRL cells cultured in FibCM. 

**Figure 2:**
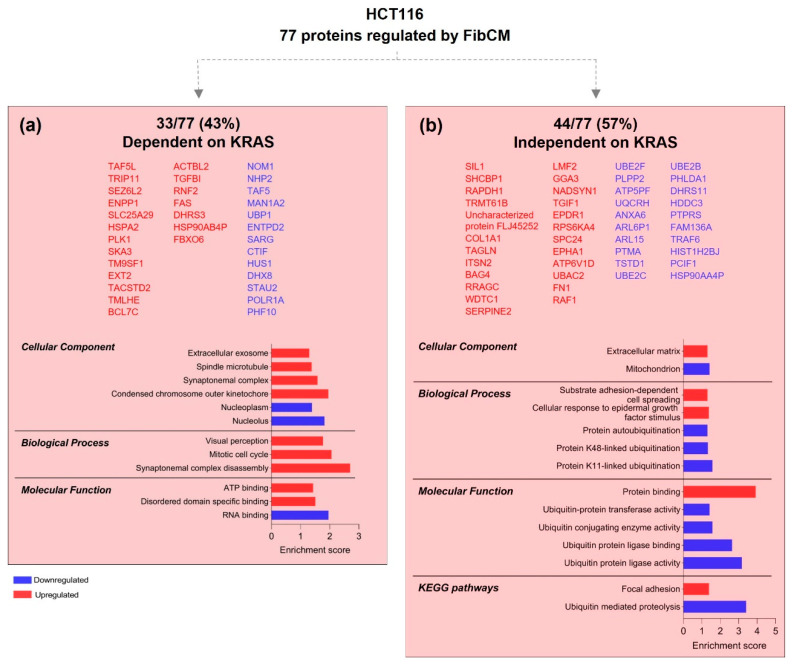
**KRAS-dependent and independent differentially expressed proteins in HCT116 cells.** GO and KEGG pathways analysis of KRAS-dependent proteins, i.e., commonly found but differentially expressed upon siKRAS (**a**), and KRAS-independent proteins, i.e., proteins exclusively found as an effect of the FibCM in siCTRL cells (siCTRL_FibCM vs. siCTRL_ctrlCM) (**b**). Protein lists are organized according to the abundance ratio. The enrichment score was calculated by—log *p*-value.

From the 77 proteins differentially expressed by HCT116 upon treatment with FibCM (Figure 1a), 33 (43%) were dependent on KRAS as they were also altered in siKRAS cells cultured with FibCM, though with different expression levels; 44 (57%) were independent of KRAS as they were exclusively found in siCTRL_FibCM (Figure 2a,b and Supplementary File S9). GO terms analysis of the 33 proteins controlled by KRAS revealed their association with the synaptomenal complex, spindle microtubule; condensed chromosome outer kinetochore, extracellular exosome, as well as with the nucleolus and nucleoplasm. Moreover, only upregulated proteins were significantly linked to synaptomenal complex disassembly, and mitotic cell cycle. At the molecular function level, both upregulated and downregulated proteins displayed binding functions: ATP and disordered domain specific, and RNA, respectively (Figure 2a). The 44 proteins exclusively identified as an effect of the FibCM, independent of KRAS, were localized at the extracellular matrix and mitochondria. Further, whereas upregulated proteins were involved in the cellular response to epidermal growth factor stimulus, substrate adhesion-dependent cell spreading, and in focal adhesion pathway; the downregulated proteins were essentially related with protein-ubiquitination processes and associated to ubiquitin-related activities (Figure 2b).

**Figure 3:**
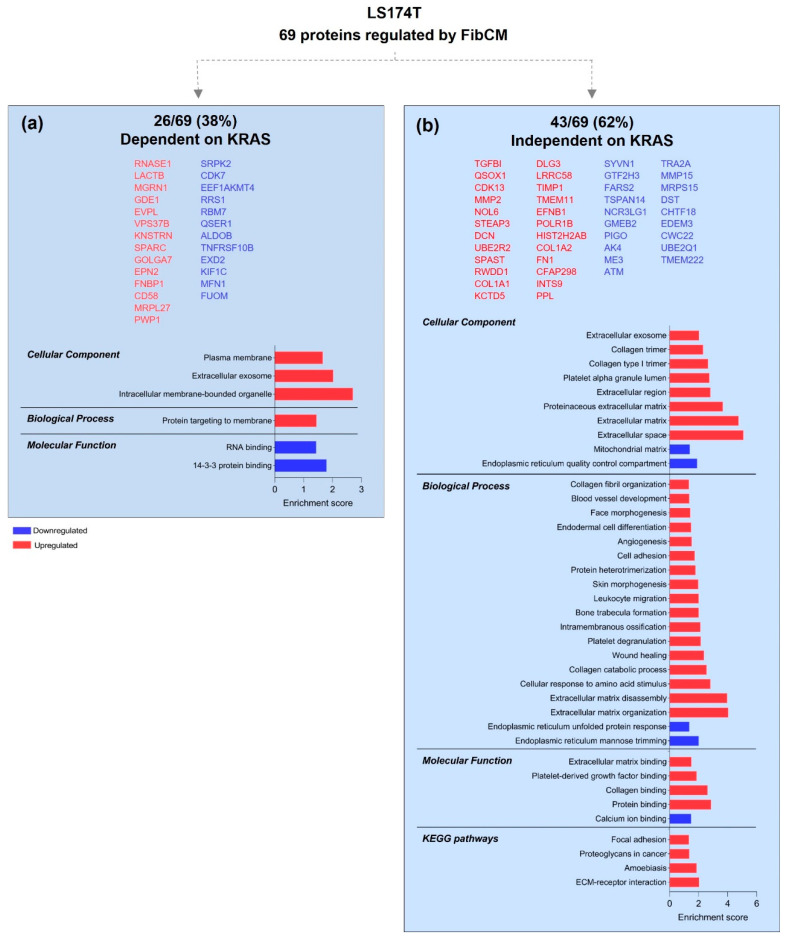
**KRAS-dependent and independent differentially expressed proteins in LS174T cells.** GO and KEGG pathways analysis of KRAS-dependent proteins, i.e., found in both conditions but differentially expressed upon siKRAS (**a**), and KRAS-independent proteins, i.e., proteins exclusively found as an effect of the FibCM in siCTRL cells (siCTRL_FibCM vs. siCTRL_ctrlCM) (**b**). Protein lists are organized according to the abundance ratio. The enrichment score was calculated by—log *p*-value.

LS174T cells differentially expressed 69 proteins in response to fibroblast-secreted factors (Figure 1c). From these, 26 (38%) were KRAS-dependent—also found in siKRAS cells cultured with FibCM, though differentially expressed; and 43 (62%) were independent of KRAS—only identified in siCTRL cells treated with FibCM (Figure 3a,b and Supplementary File S10). 

GO term analysis of the 26 proteins controlled by KRAS, revealed their association with intracellular membrane-bounded organelle, extracellular exosome, and plasma membrane as well as their involvement in processes of protein targeting to membrane (Figure 3a). Regarding the 43 KRAS-independent proteins, the upregulated ones were mainly localized at the extracellular space, extracellular exosome, ECM, and extracellular region, while the downregulated localized intracellularly at the mitochondrial matrix and the endoplasmic reticulum quality control compartment (Figure 3b). Moreover, upregulated proteins were involved in a multitude of processes, such as extracellular matrix organization and disassembly, cell adhesion, wound healing, angiogenesis and blood vessel development, collagen catabolic process and fibril organization, ECM-receptor interaction, proteoglycans in cancer and focal adhesion, whereas downregulated proteins were only involved in endoplasmic reticulum mannose trimming and endoplasmic reticulum unfolded protein response. Further, upregulated proteins displayed protein, collagen, platelet-derived growth factor and ECM binding, while the downregulated ones were involved in calcium ion binding molecular functions (Figure 3b). 

Overall, these results highlighted that from the proteome of HCT116 and LS174T modulated by fibroblasts-secreted factors, 43% and 38%, respectively, is controlled by KRAS. Both cell lines overexpressed proteins associated with extracellular exosome in a KRAS-dependent manner, whereas most of the proteins modulated by fibroblast-secreted factors independently of KRAS were involved in cell–ECM interactions.

### 3.3. The Proteomic Profile Associated with mutKRAS Is Highly Regulated by Fibroblast-Secreted Factors

Upon demonstrating that fibroblast-secreted factors modulate the proteome of CRC cells and around 40% of this proteome is dependent on KRAS, we then asked the opposite: does mutKRAS-associated protein profile change in response to external stimulation? Hence, we compared the proteome of: (i) siKRAS vs. siCTRL cells cultured in ctrlCM to unveil KRAS-dependent proteome under basal stimulation; and (ii) siKRAS vs. siCTRL cells cultured in FibCM to dissect a fibroblast-dependent mutKRAS proteome. MutKRAS-autonomous and non-autonomous protein changes were depicted by comparing these two datasets.

**Figure 4:**
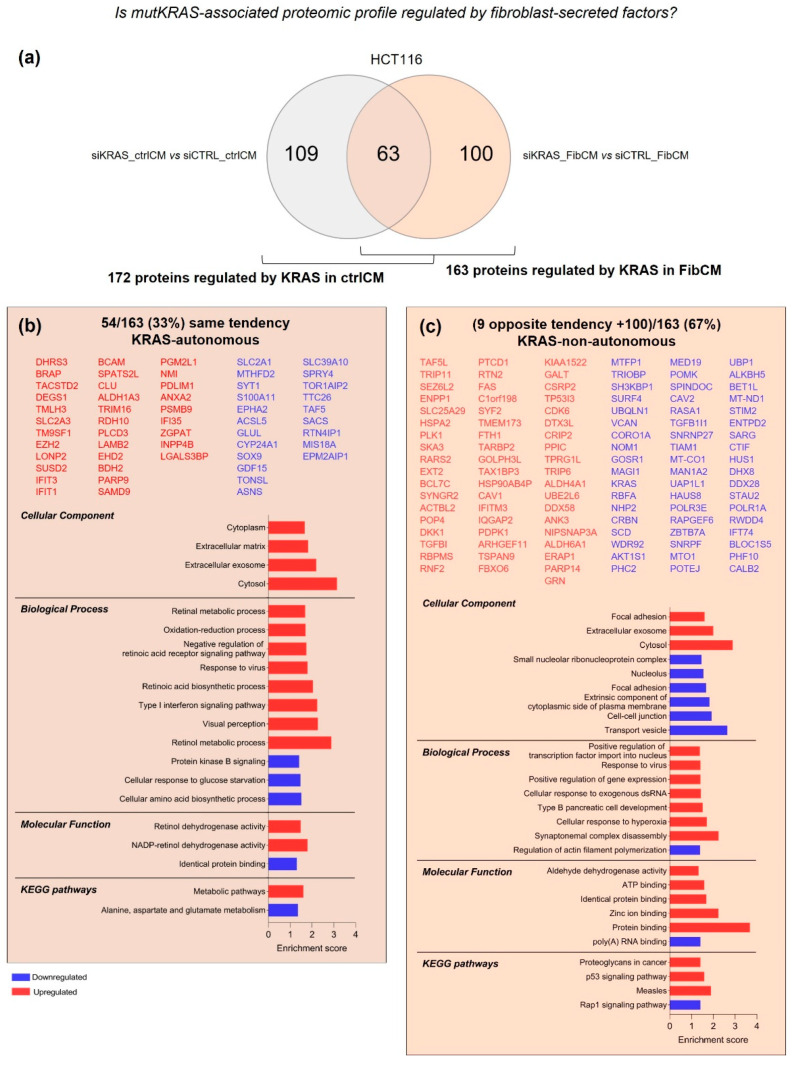
**KRAS—autonomously and non-autonomously controlled proteins in HCT116 cells**. (**a**) Venn diagram showing the number of exclusive and common proteins identified in the analyzed conditions (siKRAS_ctrlCM vs. siCTRL_ctrlCM and siKRAS_FibCM vs. siCTRL_FibCM). (**b**,**c**) GO and KEGG pathways analysis of shared proteins following the same expression tendency (KRAS-autonomous) (**b**), and shared proteins with opposite expression tendencies together with exclusive proteins from siKRAS_FibCM vs. siCTRL_FibCM comparison (KRAS-non-autonomous) (**c**). Protein lists are organized according to the abundance ratio. The enrichment score was calculated by—log *p*-value.

KRAS silencing in HCT116 cells cultured under control or FibCM stimulation altered the expression of 172 and 163 proteins, respectively (Figure 4a and Supplementary File S9). Moreover, a comparative analysis of these two datasets highlighted 63 shared proteins (Figure 4a), with 54 (54/163—33%) following the same expression tendency (either up or downregulation), meaning that they were controlled by KRAS in an autonomous way, independently of the fibroblasts-derived factors that cells were exposed to (Figure 4b). The remaining nine proteins showed opposite expression tendencies: six that were upregulated in ctrlCM (SARG, CTIF, PHF10, BLOC1S5, SPINDOC, and HUS1) became downregulated upon culture with FibCM; three (PLK1, SKA3, and RTN2) were downregulated in ctrlCM and became upregulated upon culture with FibCM (Supplementary File S9). Therefore, these nine proteins were included in the group of 100 proteins exclusively found in siKRAS cells treated with FibCM (Figure 4c and Supplementary File S9). Altogether, these 109 proteins (109/163—67%) were controlled by KRAS in a non-autonomous way, dependent on fibroblasts-derived factors.

The 54 KRAS-autonomous proteins were localized at the cytosol, extracellular exosomes, extracellular matrix, and cytoplasm, and involved in distinct biological processes, such as: retinoic acid/retinol biosynthesis and metabolism, type I interferon pathway; in amino-acid biosynthesis, response to glycose starvation, and protein kinase B signaling. Accordingly, upregulated proteins displayed NADP-retinol and retinol dehydrogenase activities and were involved in metabolic pathways, whereas downregulated proteins were involved in alanine, aspartate, and glutamate metabolism (Figure 4b). The 109 KRAS-non-autonomous proteins spanned across disperse localizations at: the cytosol, extracellular exosomes, focal adhesions, transport vesicles, cell–cell junctions, the extrinsic compartment of the cytoplasmic site of the plasma membrane, nucleolus, and at the small nucleolar ribonucleoprotein complexes. Upregulated proteins were mainly involved on synaptomenal complex disassembly, positive regulation of gene expression and transcription factors import to the nucleus as well as in proteoglycans in cancer and p53 signaling pathways. Downregulated proteins participated in actin filament polymerization biologic process and were involved in Rap1 signaling pathway. Both up and downregulated proteins displayed molecular functions of binding (Figure 4c). Overall, these results show that KRAS-silenced HCT116 cells responded to fibroblast-secretome by modulating transcription and by decreasing the expression of proteins associated with the cytoskeleton, focal adhesions, and cell–cell junctions, thus representing key proteins involved in essential cancer cell functions, such as motility.

**Figure 5:**
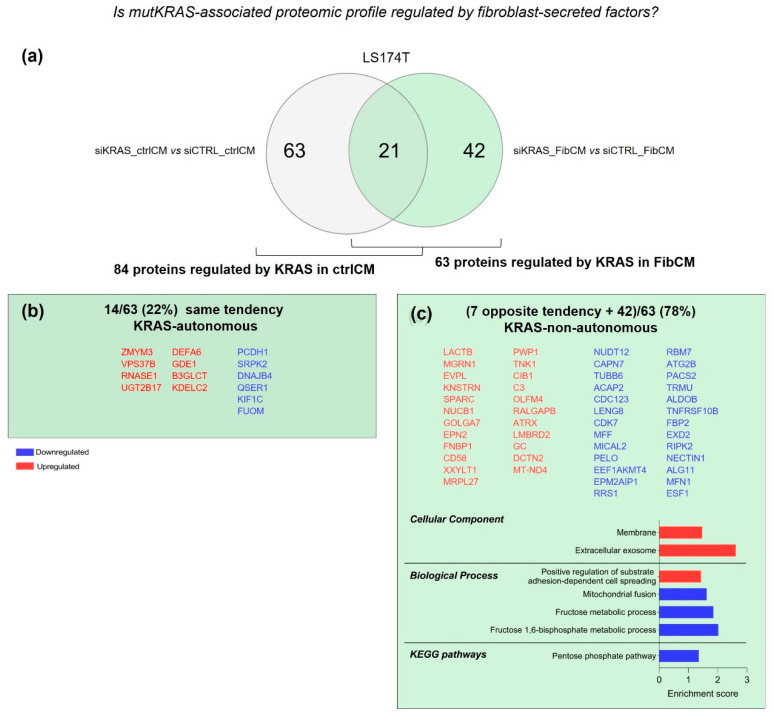
**KRAS—autonomously and non-autonomously controlled proteins in LS174T cells**. (**a**) Venn diagram showing the number of exclusive and common proteins identified in the analyzed conditions (siKRAS_ctrlCM vs. siCTRL_ctrlCM and siKRAS_FibCM vs. siCTRL_FibCM). (**b**,**c**) GO and KEGG pathways analysis of shared proteins following the same expression tendency (KRAS-autonomous) showed no significant results (**b**), and of shared proteins with opposite expression tendencies together with exclusive proteins from the comparison siKRAS vs. siCTRL cultured in FibCM (KRAS-non-autonomous) (**c**). Protein lists are organized according to the abundance ratio. The enrichment score was calculated by—log *p*-value.

The inhibition of KRAS in LS174T cells under control and FibCM stimulation resulted in the differential expression of 84 and 63 proteins, respectively. The comparative analysis of these two datasets showed 21 proteins common to both conditions (Figure 5a), from which 14 followed the same tendency (14/63—22%, KRAS-autonomous; Figure 5b) and seven followed opposite expression tendencies. These seven proteins were further included in the group of 42 proteins exclusively found in siKRAS_FibCM cells, thus revealing that 78% (49/63) of KRAS-associated proteome is dependent on fibroblast-secreted factors (Figure 5c and Supplementary File S10). 

GO analysis of the 49 KRAS-non-autonomous proteins highlighted their localization at extracellular exosomes and at the membrane and their involvement on positive regulation of substrate adhesion-dependent cell-spreading, and in fructose metabolism and mitochondrial fusion. In accordance, KEGG pathways analysis showed that downregulated proteins belong to the pentose phosphate pathway (Figure 5c). Hence, LS174T siKRAS cells likely respond to the fibroblast-secreted factors by adapting their metabolism.

Altogether, these results suggest that the proteome of HCT116 and LS174T associated to oncogenic KRAS is mainly regulated in a KRAS-non-autonomous manner, thus reinforcing the complexity and the high intrinsic and extrinsic context-dependency of mutKRAS-driven effects. 

## 4. Discussion

MutKRAS cancer cells orchestrate a pro-tumorigenic microenvironment [14,15,16,17,27]. Though, whether and how tumor microenvironment-derived signals affect mutKRAS signaling and the subsequent impact on cancer cells response has been overlooked.

Our data show that rhTGFβ1-activated fibroblasts secretome was able to differently modulate the proteome of two CRC cell lines. Several aspects may explain their distinct responses. For instance, the type of KRAS mutation: G13D in HCT116 and G12D in LS174T [28]. These mutations present biochemical and structural differences; while the G12D has low affinity to RAF and a fast hydrolysis rate, the G13D has high affinity to RAF and a more rapid GTPase activity [9]. Moreover, drug sensitivity and therapy response of these mutant forms have been shown to be different [29,30,31]. In addition, these cell lines belong to different consensus molecular subtypes (CMS)- HCT116 belongs to the CMS4, the mesenchymal type, known to be enriched in fibroblasts, while LS174T is classified as CMS3, the metabolic type [32]. In accordance, HCT116 (both siCTRL and siKRAS) were more affected by the fibroblast-derived factors; whilst in LS174T, many of the alterations found, were related with metabolic pathways. Interestingly, fibroblast-secreted factors underlined the upregulation of RAF-1 in HCT116 cells supporting the role of KRAS downstream signaling pathways in the response to microenvironmental factors. Moreover, in both cell lines, proteins related to ECM (COL1A1, FN1 and TGFBI) were found upregulated. Accordingly, TGFBI has been shown to play important roles in essential processes underlying CRC metastasis formation, such as angiogenesis [33] and extravasation [34]. In line with these effects, TGFBI expression is an independent poor prognostic factor in CRC [35]. Individually, LS174T also showed upregulation of COL1A2, MMP2, DCN, TIMP-1, and other proteins related to ECM production/remodeling. For instance, TIMP-1 was already shown to promote tumor progression in prostate and colon cancer models, by driving the accumulation of CAFs [36]. So, we demonstrated that activated-fibroblasts secretome affected important mediators of tumorigenesis, leading us to infer that it is capable to educate cancer cells towards tumor progression-related behaviors. Since both cell lines used in this study were microsatellite unstable (MSI), we speculate that the combination of mutKRAS and fibroblasts may identify a subset of MSI patients with a poor prognosis. However, further studies are needed to validate this hypothesis.

By addressing the influence of mutKRAS on the modulation of the proteome responsiveness to fibroblast-secreted factors, our data showed that 43% and 38% of the proteins modulated by fibroblast-secreted factors in HCT116 and LS174T cells, respectively, are indeed dependent on the presence of oncogenic KRAS. Given the important role of the TME derived signals to drive tumor malignant features [37], these results indicate that KRAS targeted inhibition can partially abrogate the pro-tumorigenic stimuli derived from the TME. Still, more than a half of the total proteome was found to be modulated by external factors in a KRAS-independent manner. For example, in HCT116, independently of KRAS, FibCM led to the upregulation of proteins involved in cellular response to epidermal growth factor stimulus and substrate adhesion-dependent cell spreading. In LS174T, upregulated proteins were involved in several processes such as extracellular matrix organization and disassembly, cell adhesion, wound healing, angiogenesis and blood vessel development, collagen catabolic process, and fibril organization. These data indicate that, in a KRAS-independent way, FibCM upregulated pathways that may endow cancer cells with pro-malignant features. Notably, the abovementioned biologic processes found to be mediated by fibroblasts-derived factors independently of KRAS highlight potential mechanisms of resistance to targeted therapies currently available for the treatment of CRC patients, such as anti-EGFR and anti-VEGF. More so, as KRAS-targeted therapies are becoming a reality in the clinic [5], it would be important to determine whether these KRAS-independent modifications play a role in the rapid acquisition of resistance observed in patients.

Furthermore, our results show that in some contexts, fibroblast-derived external stimuli can modulate the cancer cell proteome in a way that counteract KRAS-inhibition and support cancer cell-malignant features. While upon KRAS silencing and exposure to FibCM, HCT116 cells show a significant downregulation of Rap-1 signaling (an important signaling pathway in cancer aggressiveness, involved for instance in epithelial to mesenchymal transition, invasion, and angiogenesis promotion [38]), LS174T cells upregulate proteins that are involved in adhesion and positive cell spreading regulation. These apparent dichotomic responses go in line with our results demonstrating opposite invasive responses to fibroblast-secreted factors. While in HCT116, KRAS inhibition impaired invasion induced by fibroblasts-derived HGF, in LS174T KRAS inhibition triggered invasion [19]. Moreover, both cell lines commonly upregulated proteins associated with exosomes localization upon KRAS silencing and culture with FibCM. Since exosomes play an important role in cell-to-cell communication both locally and at distance (e.g., in metastatic niche preparation) [39,40,41], it is worth profiling the content of the exosomes produced in this context, as well as study their biological functions. 

In addition, our results show that more than 2/3 of the proteome associated to oncogenic KRAS is controlled non-autonomously by activated-fibroblasts secreted factors. In accordance, in pancreatic ductal adenocarcinoma models, mutKRAS cells have been shown to establish a non-cell-autonomous reciprocal communication with stellate cells (pancreatic fibroblasts). Importantly, sonic hedgehog-activated stellate cells secreted factors, were able to induce total proteome and phosphoproteome KRAS-non-autonomous changes, impacting cancer cell functional phenotypes [42]. It would be also interesting to evaluate the relevance of mutKRAS in mediating the response to the different subpopulations of fibroblasts that have been found in pancreatic cancer [43]. Additionally, we speculate that, in tumors, oncogenic KRAS signaling may be very heterogeneous as, cells may engage different oncogenic signaling programs according to the microenvironmental niche they are exposed to. Such context-dependency heterogeneity may dictate different responses to therapy and partially explain tumor resistance events. In fact, HCT116 KRAS silenced cells upregulated type I interferon signaling pathway in a KRAS-autonomous manner. Importantly, in CRC models, an interferon gene expression signature was shown to underlie MEK inhibition resistance in a mutKRAS context [44]. These findings highlight a possible KRAS pathway inhibition resistance mechanism, thus contributing to explain the failure of KRAS pathway inhibitors and suggesting a potential combinatorial treatment. Moreover, KRAS silencing downregulated Polo Like Kinase 1 (PLK1) in HCT116 cells. Indeed, mutKRAS cancer cells have been shown to be sensitive to PLK1 inhibition [45,46], further demonstrating PLK1 as a downstream target of mutKRAS. However, our data demonstrated that the exposure of KRAS silenced cells to FibCM rescued PLK1 downregulation, thus possibly constituting an evasion mechanism to KRAS-targeted therapies. Notably, they support PLK1 direct inhibition as a potential treatment strategy for mutKRAS patients. Accordingly, a phase 1b/2 clinical trial (NCT03829410) is currently evaluating the safety and efficacy of the PLK1 inhibitor onvansertib in combination with FOLFIRI + Bevacizumab, in mutKRAS metastatic CRC.

Overall, our data reinforce the importance of re-evaluating oncogenic KRAS signaling in the context of different TME niches as most of the proteomic profile alterations resulted from KRAS-non-autonomous signaling. This will certainly advance the understanding of mutKRAS CRC biology and empower the identification of novel treatment strategies based on targeting the crosstalk between mutKRAS cancer cells and the TME. Nonetheless, the identification of a KRAS-autonomous signature common to different types of stimuli is likely to reveal valuable actionable targets worthy to impair mutKRAS cells irrespective of their microenvironment-induced signaling heterogeneity. 

## 5. Conclusions

This work constitutes a step forward towards the understanding of KRAS-associated signals in response to microenvironmental cues. Moreover, it highlights the need of the development of more complex models that can faithfully mimic the complexity of the TME to study the behavior of mutKRAS cancer cells and generate translational knowledge to be used in the identification of therapeutic targets and biomarkers of resistance to therapy. 

## Data Availability

The mass spectrometry proteomics data have been deposited to the ProteomeXchange Consortium via the PRIDE [23] partner repository with the dataset identifier PXD030551 and 10.6019/PXD030551. The rest of the data generated in this work is contained within the article or supplementary material.

## Supplementary Materials

The following supporting information can be downloaded at: https://www.mdpi.com/article/10.3390/cells11131988/s1. Figure S1: Representative Western blot showing increased expression of α-SMA following CCD-18Co fibroblasts activation with rhTGFβ1; Figure S2: Representative Western blots showing efficient KRAS silencing in HCT116 and LS174T cell lines; Supplementary File S1: HCT116_all_identified_proteins; Supplementary File S2: LS174T_all_identified_proteins; Supplementary File S3: HCT116_siCTRL_FibCM_vs_siCTRL_ctrlCM; Supplementary File S4: LS174T_siCTRL_FibCM_vs_siCTRL_ctrlCM; Supplementary File S5: HCT116_siKRAS_FibCM_vs_siCTRL_FibCM; Supplementary File S6: HCT116_siKRAS_ctrlCM_vs_siCTRL_ctrlCM; Supplementary File S7: LS174T_siKRAS_FibCM_vs_siCTRL_FibCM; Supplementary File S8: LS174T_siKRAS_ctrlCM_vs_siCTRL_ctrlCM; Supplementary File S9: Lists of differentially expressed proteins in HCT116 cell line_all comparisons; Supplementary File S10: Lists of differentially expressed proteins in LS174T cell line_all comparisons.
