# Supplementary material for: Mutant KRAS-Associated Proteome Is Mainly Controlled by Exogenous Factors
Source: Cells. 2022 Jun 21;11(13):1988. doi: 10.3390/cells11131988 (PMC9265670; doi:10.3390/cells11131988)
Supplement: Supplementary file 1 [file cells-11-01988-s001.zip › Supplementary figures.pdf]

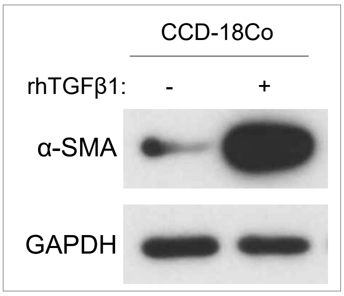

Figure S1: Representative western blot showing increased expression of α-SMA following CCD-18Co fibroblasts activation with rhTGFβ1.

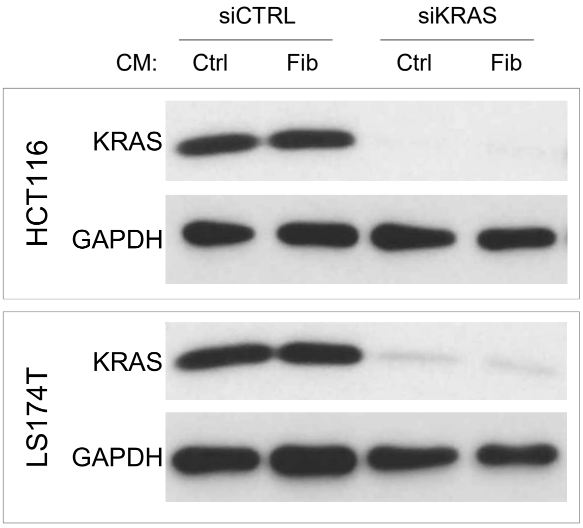

Figure S2: Representative western blots showing efficient KRAS silencing in HCT116 and LS174T cell lines.
